# Guided internet-based cognitive behavioral therapy for insomnia in patients with borderline personality disorder: Study protocol for a randomized controlled trial

**DOI:** 10.1016/j.invent.2022.100563

**Published:** 2022-07-21

**Authors:** S. van Trigt, T. van der Zweerde, E.J.W. van Someren, A. van Straten, H.J.F. van Marle

**Affiliations:** a Amsterdam UMC, location Vrije Universiteit Amsterdam, Psychiatry, De Boelelaan 1117, Amsterdam, the Netherlands; b Amsterdam Public Health, Mental Health Program, Amsterdam, the Netherlands; c GGZ inGeest Mental Health Care, Amsterdam, the Netherlands; d Amsterdam Neuroscience, Mood, Anxiety, Psychosis, Sleep & Stress Program, Amsterdam, the Netherlands; e Department of Sleep and Cognition, Netherlands Institute for Neuroscience, an Institute of the Royal Netherlands Academy of Arts and Sciences, Amsterdam, the Netherlands; f Department of Integrative Neurophysiology, Center for Neurogenomics and Cognitive Research, Amsterdam Neuroscience, Vrije Universiteit, Amsterdam, the Netherlands; g Vrije Universiteit Amsterdam, Clinical, Neuro and Developmental Psychology, Amsterdam, the Netherlands

**Keywords:** Borderline personality disorder, Insomnia, Sleep, Cognitive behavioral therapy for insomnia, Internet intervention, Randomized controlled trial

## Abstract

Borderline personality disorder (BPD) is a highly disabling psychiatric disorder with emotion dysregulation at its core, resulting in affective instability, impulsivity and sometimes self-harming or suicidal behavior. Sleep is increasingly recognized to play a crucial role in emotion regulation. BPD patients often suffer from (severe) insomnia, potentially aggravating symptoms and preventing recovery from BPD. Yet, the effects of insomnia treatments have not been investigated in context of BPD. Guided internet-based cognitive behavioral therapy for insomnia (iCBT-I; i-Sleep) has been proven effective in improving both insomnia and affective symptoms. In this randomized controlled trial among 96 patients with a DSM-5 diagnosis of BPD (or other personality disorder with ≥4 BPD traits) and insomnia symptoms, we will test the effectiveness of iCBT-I before regular BPD treatment starts, during the waitlist period, on BPD symptoms. Patients in the control group monitor their sleep through a sleep diary during the waitlist period and also receive standard BPD treatment after that. Using linear mixed models we will test the hypothesis that the iCBT-I group improves more than the control group on BPD symptoms (primary outcome), insomnia severity, additional subjective and objective sleep variables, emotion regulation, comorbid anxiety and depression complaints, and quality of life. These effects are thought to arise from a direct effect of improved sleep on emotion regulation and a synergistic effect on the consolidation and internalization of the BPD treatment effect. To our knowledge, this is the first trial assessing effectiveness of CBT-I in patients with BPD (traits). The accessibility of the studied intervention greatly facilitates clinical implication in case of positive results.

## Highlights

• Emotion dysregulation is at the core of borderline personality disorder (BPD).
• Healthy emotion regulation requires adequate sleep.
• Yet BPD patients often suffer from sleep disturbances, such as insomnia.
• This is the first RCT studying the effect of online sleep therapy in BPD patients.
• The accessibility of the intervention facilitates future clinical implementation.

## 1 Introduction

### 1.1 Background

Borderline personality disorder (BPD) is a severe psychiatric disorder with prevalence rates of 1–5 % in the general population, and even 15–28 % in clinical populations (Gunderson et al., 2018). BPD is characterized by an enduring and pervasive pattern of instability in interpersonal relationships, self-image and affects, and marked impulsivity and self-harming and suicidal behaviors (American Psychiatric Association, 2013). Patients with BPD suffer from both emotional dysregulation (e.g., highly reactive mood and emotions, anger attacks, feelings of emptiness, and fear of abandonment), as well as behavioral dysregulation (e.g., impulsive, suicidal, and self-harming behaviors) and cognitive disturbances (identity disturbances, paranoid ideation, and dissociation) (Leichsenring et al., 2011). Given the associated functional impairment and extensive use of (mental) health care, BPD is associated with considerable personal and societal burden, constituting an important public health problem (Juurlink et al., 2018; Juurlink et al., 2020).

Emotion dysregulation lies at the core of all BPD symptomatology (Gunderson et al., 2018). A growing literature highlights the essential role of sleep in emotion regulation and emotional memory processing (Goldstein and Walker, 2014; Rasch and Born, 2013; van der Helm and Walker, 2011). Healthy emotion regulation is thought to depend critically on solid, undisturbed sleep. In contrast, recent studies have indicated that people with insomnia may suffer from fragmented rapid eye movement (REM)-sleep (frequent transitions from REM-sleep to wake and light sleep), which related to a failure of overnight alleviation of emotional distress and amygdala reactivity (Wassing et al., 2019a; Wassing et al., 2019b). Furthermore, sleep is crucial for memory processing (Goldstein and Walker, 2014; Rasch and Born, 2013). During a process referred to as memory consolidation, newly acquired or updated memories are integrated in existing cortical memory networks, stabilizing them and warranting their long-term retention (Dudai et al., 2015; Frankland and Bontempi, 2005). The consolidation of emotional memories is thought to depend primarily on (intact) REM-sleep (van der Helm and Walker, 2011; Nishida et al., 2009). Adequate sleep may therefore be considered imperative for successful psychological treatment, since adjusted dysfunctional memories and newly acquired behavioral and cognitive skills need to be consolidated for psychiatric symptoms to subside (Lane et al., 2015).

Disturbed sleep is thus a highly important factor in BPD severity and recovery. BPD patients often experience severe sleep disturbances (DeShong and Tucker, 2019; Schredl et al., 2012; Vanek et al., 2021). A recent meta-analytic review showed both subjective (e.g. lower sleep quality) and objective (e.g. difficulty falling asleep, reduced sleep efficiency, less deep sleep and higher REM-density) sleep disturbances in BPD (Winsper et al., 2017). Some of these disturbances aggravate emotion dysregulation and self-harming and suicidal behavior in BPD (DeShong and Tucker, 2019; Winsper et al., 2017). Insomnia is the most common of all sleep disorders, characterized by difficulties with initiating sleep, maintaining sleep during the night, and/or waking too early from sleep (American Psychiatric Association, 2013). Up to 63 % of BPD patients report having at least one insomnia symptom nearly every night (Selby, 2013), making insomnia a highly prevalent complaint in BPD.

Until recently, insomnia was most often seen as a consequence or symptom of other psychiatric disorders. Yet, insomnia is increasingly considered a causal factor in development and maintenance of affective psychiatric disorders, because of the centrality of sleep in emotion regulation and emotional memory processing. However, studies assessing the relation between BPD and sleep are scarce. Controlled studies focusing on the role of sleep in BPD treatment are nonexistent (Winsper et al., 2017; Hafizi, 2013). Although effectiveness of sleep therapy in BPD populations is not yet assessed, there is solid meta-analytic evidence that cognitive behavioral therapy for insomnia (CBT-I) is effective also in people with psychiatric disorders, such as depression and post-traumatic stress disorder (PTSD) (Geiger-Brown et al., 2015; van Straten et al., 2018; Wu et al., 2015; Ho et al., 2016). CBT-I commonly consists of three elements: (1) information (psychoeducation, sleep hygiene), (2) behavioral exercises (stimulus control, sleep restriction, relaxation), and (3) cognitive exercises (tackling dysfunctional thoughts about sleep) (Riemann et al., 2017). In order to increase availability and accessibility of CBT-I, guided and unguided internet-based versions have been developed: iCBT-I. A recent meta-analysis showed the effectiveness of iCBT-I for insomnia (Zachariae et al., 2016). Within our group we developed the guided online iCBT-I intervention called i-Sleep. This intervention was tested in four RCT's covering different populations with insomnia complaints, showing large effect sizes on self-reported quality of sleep, sleep-diary derived sleep continuity and secondary outcomes such as symptoms of depression (van Straten et al., 2013; van der Zweerde et al., 2020; van der Zweerde et al., 2018; Leerssen et al., 2021). Adding CBT-I to standard treatment in patients suffering from BPD and insomnia complaints may be promising, not only to reduce sleep disturbances, but also to mitigate BPD symptoms. Yet, the effectiveness of CBT-I has not yet been tested in BPD patients.

### 1.2 Objectives

This randomized controlled trial (RCT) investigates the effectiveness of adding guided iCBT-I to standard BPD treatment focusing on emotion dysregulation in patients with BPD or another personality disorder (PD) with ≥4 BPD traits (at least one typical emotional dysregulation symptom) and insomnia complaints before their regular BPD treatment takes place. During the same period, patients in the control group will monitor their sleep in sleep diaries before their regular BPD treatment takes place. We hypothesize that patients who receive guided iCBT-I prior to BPD treatment will improve more than the control group on BPD symptoms (primary outcome), insomnia severity, subjective and objective sleep disturbances (as measured with sleep diary and EEG headband), emotion regulation, comorbid anxiety and depression symptoms, quality of life and daytime functioning (secondary outcomes). We expect that BPD symptoms will improve directly following iCBT-I (a direct effect of improved sleep on emotion regulation) and even further after standard BPD treatment (a synergistic effect of improved sleep on the consolidation and internalization of the treatment effect).

## 2 Methods

### 2.1 Study design

We will perform a two-armed RCT. In the experimental arm, patients will receive guided iCBT-I for 5–8 weeks followed by a standard BPD treatment (Systems Training for Emotional Predictability and Problem Solving, STEPPS) (Blum et al., 2008). In the control arm, patients are on the waitlist and keep a sleep diary for 6 weeks, followed by STEPPS. The outcomes will be assessed by clinical interviews, questionnaires, sleep diary and EEG headband at baseline (T0), after iCBT-I/waitlist (T1 at 2 months), and after STEPPS (T2 at 8 months). The study design was approved by the Medical Ethics Committee of the VU Medical Centre (METC VUmc; NL76232.029.20). The trial is registered with the Netherlands Trial Register (NL9776).

### 2.2 Study population

The study population consists of patients with a DSM-5 diagnosis of BPD or other PD with ≥4 BPD traits, recruited from outpatient departments for personality disorders at specialized mental health care center GGZ inGeest in the Netherlands. All participating patients are registered with the mental health care center and assigned to a psychologist or psychiatrist. In case of crisis or requests for assistance unrelated to sleep, patients are referred to their assigned therapist. All patients with BPD (traits) on the STEPPS-waitlist are potentially eligible participants. For inclusion patients should meet the following criteria: (1) BPD diagnosis, or other PD diagnosis with ≥4 BPD traits (at least one typical emotion dysregulation trait: affective instability, impulsivity, parasuicidal behavior, or anger attacks) according to DSM-5 criteria, as assessed with the Structured Clinical Interview for DSM-5 Personality Disorders (SCID-5-P) (First et al., 2017), (2) 18–65 years, (3) sleep complaints (a score of ≥10 on the Insomnia Severity Index (ISI) (Bastien et al., 2001)), (4) self-considered capability of completing online questionnaires and diaries in Dutch. Exclusion criteria are mostly based on the probable inability to comply with iCBT-I instructions: (1) current diagnosis of bipolar or psychotic disorder, as assessed with a self-report yes/no question, (2) alcohol or drug dependency (a score of ≥20 on the Alcohol Use Disorders Identification Test (AUDIT) (Saunders et al., 1993) or ≥25 on the Drug Use Disorder Identification Test (DUDIT) (Berman et al., 2005)), and (3) having received CBTI in the past 3 months. Using sleep medication is allowed. Patients with sleep disorders other than insomnia (e.g., sleep apnea, narcolepsy) are allowed to participate to enhance generalizability of our results.

### 2.3 Interventions

#### 2.3.1 iCBT-I

The guided, internet-delivered CBT-I intervention i-Sleep was developed and later updated by VU Amsterdam (van Straten et al., 2013; van der Zweerde et al., 2016). It consists of five sessions: (1) Psycho-education on sleep, disordered sleep and sleep hygiene (i.e. guidelines about health and environmental factors influencing sleep), (2) stimulus control training (i.e. use the bed for sleep only to reinforce association of bed with sleeping) and sleep restriction therapy (i.e. restrict time in bed to average time slept), (3) relaxation techniques and minimizing worrying, (4) tackling dysfunctional cognitions about sleep, and (5) summary and relapse prevention plan (van der Zweerde et al., 2020). See Supplementary material for a more detailed description of the intervention. Patients fill out a sleep diary during the entire treatment period. The treatment takes 5–8 weeks to complete, and is offered online using text and videos. I-Sleep includes experience-based case vignettes of patients. We use an adapted mental health care version of i-Sleep in which case vignettes represent patients with comorbid psychiatric symptoms. Reading the session text and carrying out the homework exercises of i-Sleep take approximately 10–30 min daily. After every session the therapist provides online feedback, guides sleep restriction therapy and bedtimes, and motivates patients to persevere in treatment. Patients can also send questions to their therapist. In general, guidance takes about 20 min per patient per session. There are three additional 30-minute video call appointments (at the start, and after 2 and 4 weeks) to provide extra explanation, enhance commitment, and prevent drop-out. In this study the main researcher will provide the feedback (SvT). She is trained by a psychologist with extensive experience in CBT-I and i-Sleep (TvdZ) and under weekly supervision by TvdZ and another experienced CBT(-I) psychologist. I-Sleep will be delivered through the secure web-based platform the Nederlands Slaap Register (www.slaapregister.nl).

#### 2.3.2 STEPPS

STEPPS is a 20-week manual-based group treatment for patients with BPD symptoms (Blum et al., 2008). STEPPS is a cognitive-behavioral skills training approach characterizing BPD as a disorder of emotion and behavior regulation. STEPPS aims to improve emotion regulation by becoming more aware of thought patterns, feelings, and behaviors that define BPD, and to acquire emotion and behavior management skills. Patients receive individual treatment parallel to group treatment, mostly to guide homework exercises and provide personal guidance. STEPPS is delivered by trained clinicians working at GGZ InGeest. At some locations of the trial a shortened 10-week version of STEPPS is offered, which is a practice-based adaptation of the 20-week version, including the same goals and core features (Freije, 2016). STEPPS is tested in several RCTs showing moderate to large effects sizes on BPD symptoms (Blum et al., 2008; Bos et al., 2010).

### 2.4 Study procedure

**Fig. 1:**
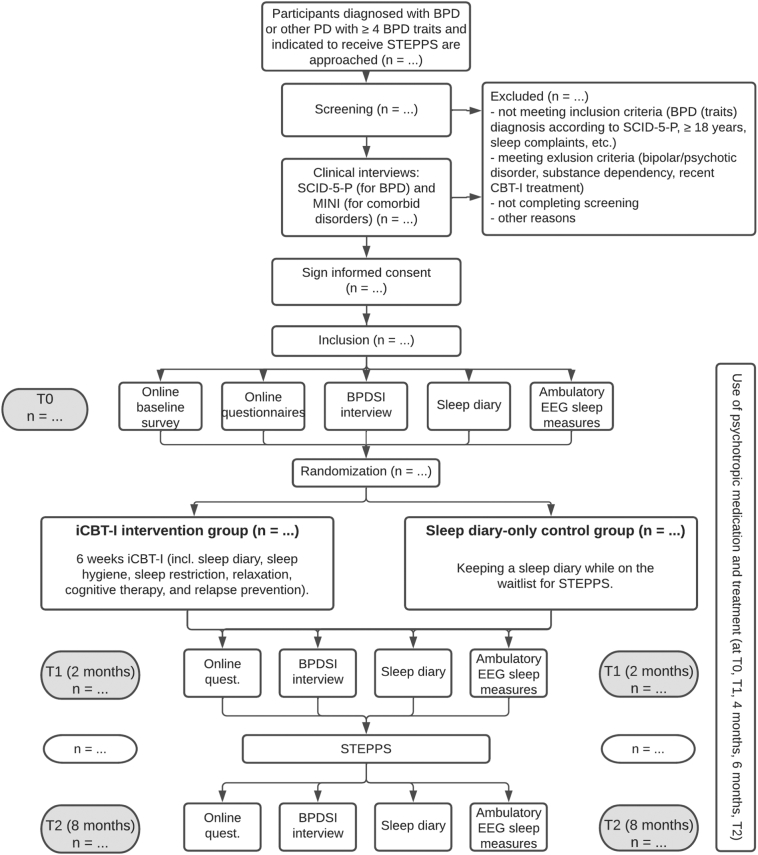
Flowchart of study procedures. Note. BPD: borderline personality disorder; BPDSI: Borderline Personality Disorder Severity Index; iCBT-I: internet-based cognitive behavioral therapy for insomnia; SCID-5-P: Structured Clinical Interview for DSM-5 Personality Disorders; STEPPS: Systems Training for Emotional Predictability and Problem Solving.

**Fig. 2:**
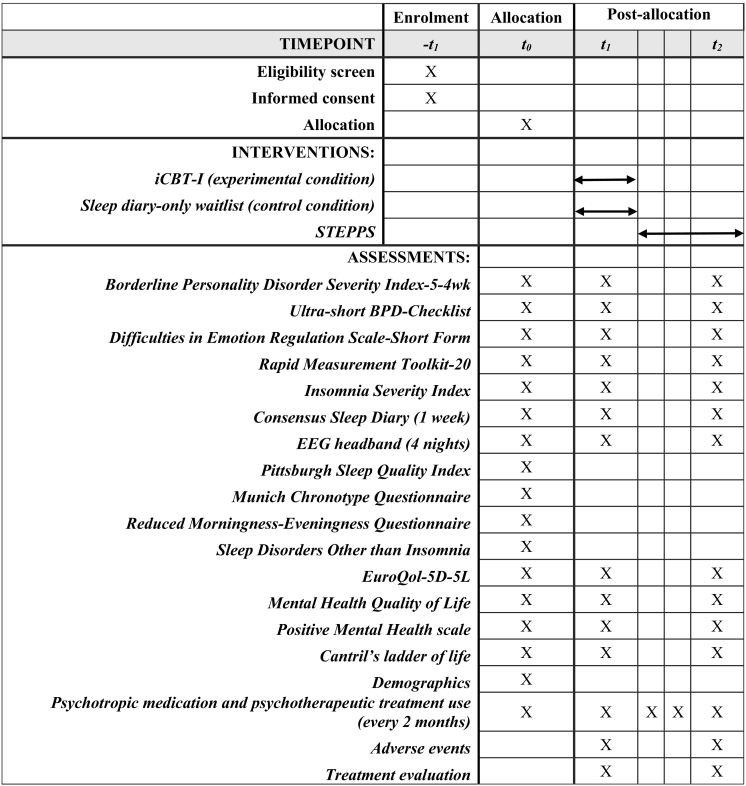
Schedule of enrolment, interventions, and assessments. Note. T1 and T2 are 2 months and 8 months after baseline respectively. Use of medication and treatment is additionally assessed between T1 and T2; at 4 months and 6 months.

A flowchart of the study procedure is displayed in Fig. 1. Fig. 2 shows an additional overview of all assessments and interventions. Patients are recruited through their treating physicians at GGZ InGeest. Patients on the STEPPS-waitlist that provided consent are contacted by the researchers by phone. The researchers then provide information and send the participant information folder and informed consent (IC) by email. If interested in participation, an appointment is scheduled for screening and administering the SCID-5-P, a 119-item semi-structured interview for diagnosing PDs according to DSM-5 criteria (First et al., 2017), to confirm BPD diagnosis and traits. Based on the SCID-5-P screener, other sections may be administered to diagnose possible comorbid PDs. In case the SCID-5-P interview is completed already within the last 6 months before inclusion, these data can be copied from the patient's medical file. Possible comorbid psychiatric disorders are assessed with the Mini-International Neuropsychiatric Interview (MINI) (Sheehan et al., 1998). If eligible, patients are invited to ask questions and sign IC.

Subsequently, patients commence with the T0 assessment, which contains all outcome measures and an online baseline survey. All outcome assessments are repeated at T1 (2 months after T0, after either iCBT-I and ideally before STEPPS starts, or waitlist) and T2 (8 months after T0, after STEPPS). Use of psychotropic medication and psychotherapeutic treatment is additionally assessed at 4 and 6 months after T0. After T0, patients are randomly assigned to iCBT-I group or the sleep diary-keeping control group. During all phases of the trial, there is one central contact person to provide support to all patients. After completion of the trial, patients in the control condition will be offered identical treatment of remaining sleep problems.

Patients may withdraw from participation at any time, for any reason, without consequences for their awaited treatment. We aim to perform intention-to-treat analyses, so patients that decide to withdraw from treatment are asked to continue the outcome assessments. We will continue recruitment until the intended sample size is achieved or until funding restrictions compel us to end assessments. Data of patients that withdraw from participating are used and reported, unless patients ask us to destroy any data.

### 2.5 Outcomes and measurements

#### 2.5.1 Primary outcome measure

The primary outcome is BPD severity, as assessed with the BPDSI-5-4wk. This clinician-rated, semi-structured interview is administered at T0, T1, and T2. The BPDSI-5-4wk is an adjusted version of the BPDSI-5 and measures BPD symptom severity over a period of the past 4 weeks instead of 3 months (Arntz and Wibbelink, 2019). The BPDSI-5-4wk is based on the DSM-5 criteria for BPD and consists of 70 items assessing behaviors and cognitions related to abandonment, relationships, self-image, impulsivity, (para)suicidal behavior, mood swings, feelings of emptiness, anger attacks, and dissociation and paranoid ideation on an 8-point scale ranging from 0 to 10. Scoring options of the BPDSI-5 are adjusted for the BPDSI-5-4wk to (0) never/4 weeks, (3) once/4 weeks, (5) twice/4 weeks, (6) three times/4 weeks, (7) once a week, (8) multiple times a week, but less than half of the week, (9) more than half of the week, and (10) daily. Note that scores 1, 2, and 4 have been omitted, to obtain the same frequency to time ratio used in the BPDSI-5. The BPDSI has high interrater reliability, acceptable to high internal consistencies, and very good discriminant and concurrent validity (Giesen-Bloo et al., 2010).

#### 2.5.2 Secondary outcome measures

##### 2.5.2.1 Self-reported BPD severity

BPD severity is additionally assessed using a self-report questionnaire, the BPD Checklist – ultra-short (ultra-short BPD-C) (Bloo et al., 2017; Wibbelink and Arntz, 2020). This additional measure allows us to combine data with parallel studies assessing the effectiveness of iCBT-I in other psychiatric populations. The ultra-short BPD-C consists of nine items rated on a scale of 1 (not at all) to 5 (very much), one for each DSM-5 BPD diagnostic criterion, with higher sum scores indicating higher BPD severity. The ultra-short BPD-C has good internal consistency, and good convergent, discriminant, and construct validity (Wibbelink and Arntz, 2020).

##### 2.5.2.2 Insomnia severity

Insomnia severity is measured with the ISI (Bastien et al., 2001). The ISI has seven items rated on a scale of 0 to 4, with higher sum scores indicating more severe insomnia. The ISI has adequate internal consistency, is sensitive to change, and has been validated for online use (Thorndike et al., 2011; Morin et al., 2011).

##### 2.5.2.3 Subjective sleep (sleep diary)

Patients keep a sleep diary that is based on the Consensus Sleep Diary (CSD) (Carney et al., 2012) for 1 week to establish the following sleep parameters: sleep onset latency, sleep efficiency, number of awakenings, wake after sleep onset, terminal wakefulness, total sleep time, and subjective sleep quality. Patients also rate how energized, depressed, anxious, and distressed they feel on a scale of 0 (not at all) to 4 (very much) each morning and optionally also in the evening. Patients are asked to fill out the sleep diary every morning, preferably 30–60 min after awakening. Note that sleep diary data that is collected between T0 and T1 as part of either iCBT-I or the sleep diary control condition will not be used as outcome measure.

##### 2.5.2.4 Objective sleep (EEG headband measurements)

To measure sleep objectively, patients are asked to sleep with an EEG headband (ZMax, Hypnodyne) for four nights at each assessment. The ambulatory EEG headband, which is easy to use and comfortable, is send to the patient's home with clear instructions for use. Sleep parameters that are derived from EEG measurements concern sleep continuity measures (e.g. sleep efficiency and number of awakenings) as well as sleep architecture measures (e.g. duration and percentage of the different sleep stages and REM-fragmentation). EEG assessments are optional and patients are not excluded if they are unwilling to sleep with the headband.

##### 2.5.2.5 Emotion regulation

Emotion regulation is measured with the Difficulties in Emotion Regulation Scale – Short Form (DERS-SF) (Kaufman et al., 2016). The DERS-SF consists of 18 items, rated on a scale from 1 (almost never) to 5 (almost always), loading onto six subscales: (1) lack of emotional awareness, (2) lack of emotional clarity, (3) difficulty regulating impulsive behavior, (4) difficulty engaging in goal-directed behaviors, (5) non-acceptance of emotional responses, and (6) lack of access to emotion regulation strategies. Total scores range from 18 to 90 with higher scores indicating more difficulties in emotion regulation. The DERS-SF has good internal consistency and construct validity (Hallion et al., 2018; Skutch et al., 2019). However, because the psychometric soundness of the DERS-SF subscale covering emotional awareness is lacking (Hallion et al., 2018; Moreira et al., 2020), these questions are replaced by questions of the DERS-18 (Victor and Klonsky, 2016), in line with the procedure in Wibbelink et al. (2022).

##### 2.5.2.6 Severity of anxiety and depression symptoms

Symptoms of generalized anxiety disorder (GAD), social anxiety disorder (SAD), panic disorder (PD), PTSD and major depressive disorder (MDD) are assessed using the Rapid Measurement Toolkit-20 (RMT-20) (Batterham et al., 2020). The RMT-20 is a self-report questionnaire with five times four items, rated on a scale of 1 (never) to 5 (always), assessing the severity of each disorder. Higher scores indicate higher anxiety or depression severity. The RMT-20 has high internal consistency and excellent sensitivity and specificity (Batterham et al., 2020).

##### 2.5.2.7 Additional outcomes

Additional outcome measures consist of quality of life, daytime functioning, positive mental health, and life satisfaction outcomes (see Supplementary material for description).

#### 2.5.3 Baseline measurements

Several baseline measurements are obtained through online survey and used for covariate-adaptive randomization, population description and effect modification analyses. Demographic data include sex, age, nationality, height and weight, employment, and education. Questions regarding use of psychotropic medication and psychotherapeutic treatment are repeated every 2 months until T2. Additionally, sleep quality, chronotype, and symptoms of sleep disorders other than insomnia are assessed at baseline (see Supplementary material).

#### 2.5.4 Adverse effects

Potential adverse effects are assessed in two ways, in line with procedures in van der Zweerde et al. (2016). First by assessment of BPD and insomnia severity. Exacerbation of BPD or insomnia severity from pre- to post-iCBT-I may indicate adverse effects. Second, patients report at T1 and T2 whether they experienced (1) falling accidents, (2) traffic accidents, and (3) any other negative events they think are related to sleepiness or fatigue.

#### 2.5.5 Treatment evaluation

Patients in the intervention condition are asked to evaluate the iCBT-I treatment and its guidance at T1 and T2 (see Supplementary material).

### 2.6 Randomization

To guarantee blindness during baseline diagnostic interviews, patients are randomized only after T0 assessments, by an independent researcher. Simple randomization is applied for the first 10 patients. To ascertain balanced, equally sized groups, subsequent patients are assigned using covariate adaptive randomization (group size, sex, age, use of psychotropic medication (current/past/never), time of year of inclusion (week number), baseline severity of BPD (BPDSI and BPD-C scores), insomnia (ISI scores), depression and anxiety (RMT-20 scores), duration of STEPPS (10 or 20 weeks), and diagnosis (BPD or other PD with ≥4 BPD traits)) scripted in R and carried out by an independent researcher. Covariate adaptive randomization is a minimization randomization method which minimizes imbalance on selected factors (covariates) and is in line with the CONSORT statement, which describes this method as equivalent to general randomization (Schulz et al., 2010). Due to the nature of the intervention, the therapist is not blind to treatment condition. The primary outcome measurement (BPDSI) is administered by a blinded and trained researcher. In addition, EEG data is scored by a trained researcher who is blind to experimental condition. All other outcome assessments involve self-report questionnaires that remain blinded. For analysis, all data is re-coded regarding participant number and condition to ensure blinding.

### 2.7 Sample size calculation

A recent meta-analysis assessing the effect of CBT-I in patients with insomnia and comorbid psychiatric disorders (MDD, PTSD, and alcohol and hypnotic dependence), found medium to large effect sizes (*d* ≥ 0.5) on psychiatric symptoms (Wu et al., 2015). This meta-analysis contains both studies with an active (e.g., sleep diary, sleep hygiene only) or inactive (e.g., waiting list, care as usual) control condition. Using linear mixed models to estimate treatment effects averaged over T1 and T2, based on a medium effect size (Cohen's *d* = 0.5) on our primary outcome BPD severity, α = 0.05, power = 0.80 and correlation among repeated measures = 0.5, 48 patients per condition are needed.

### 2.8 Analyses

For all outcomes, we will fit linear mixed models to estimate effects of time and the interaction between time and treatment allocation, while omitting the main effect of treatment allocation to control for baseline differences. The outcome measures are structured hierarchically because of the repeated measures nested within subjects. The effect of interest for establishing the effectiveness of iCBT-I is represented by the two time by treatment allocation terms. In post-hoc analysis we will analyze whether the effect on BPDSI score mainly results from iCBT-I alone (comparing T0 with T1) or the combination of iCBT-I and STEPPS (comparing T0 with T2 and adding measurements at T1 as moderators). Possible effect modification of individual differences in several mental health predictors (baseline severity of insomnia, anxiety, and depression symptoms) are assessed by adding them as predictors together with their interaction with treatment allocation. We will conduct intention-to-treat analyses. Assumptions underlying our planned analyses will be checked and reported. Sleep diary data and EEG data will both be aggregated into weekly and 4-night averages respectively. Analyses will be carried out in R using α = 0.05 for all significance tests.

## 3 Discussion

Severe emotion dysregulation lies at the core of BPD, resulting in typical symptoms such as affect instability, fear of abandonment and impulsive or self-harming behavior. Healthy emotion regulation depends critically on solid, undisturbed sleep, yet sleep disturbances such as insomnia are extremely common in BPD. By improving sleep through guided iCBT-I prior to emotion regulation treatment, we aim to improve sleep as well as mitigate BPD symptoms. Better sleep may promote more efficient processing and overnight alleviation of (negative) emotions accumulated during the day, as well as affective stability the next day. On a symptom level this may result in fewer mood swings, inappropriate anger attacks, intense feelings of emptiness and fears of abandonment. Emotional dysregulation also drives the severe behavioral and cognitive symptoms in BPD. We therefore expect better sleep to similarly result in less self-damaging or suicidal behavior, impulsiveness and paranoid ideation. Apart from these direct effects, we expect that optimizing sleep with iCBT-I prior to standard BPD treatment will also facilitate (memory) consolidation of the treatment outcome. Newly acquired emotion regulation skills and adjusted dysfunctional beliefs about self and others will be more strongly internalized, further reducing BPD symptom severity. For secondary outcomes we expect an improvement in insomnia complaints, subjective and objective sleep disturbances (sleep diary and EEG headband), emotion regulation, anxiety and depressive symptoms, and quality of life and daytime functioning. Our ultimate goal is to test the efficacy of iCBT-I as add-on therapy for BPD with comorbid sleep complaints and include the intervention in clinical guidelines of BPD in case of positive results. Findings may similarly spark new investigations into effectiveness of iCBT-I for other psychiatric disorders deriving from emotion dysregulation and/or maladaptive memory processing along with sleep problems.

### 3.1 Strengths and limitations

The study has several strengths. First, administering iCBT-I prior to standard BPD treatment allows us to study the direct effect of iCBT-I on BPD symptomatology (at T1) and the synergistic effect with standard emotion regulation therapy (at T2). Second, a particular methodological strength is the vast variety of thoroughly assessed outcome measures. BPD severity is assessed through clinical interview as opposed to mere self-report. In addition, sleep parameters are assessed both subjectively and objectively, including physiological sleep measures through ambulatory EEG. This allows for an in-depth analysis of sleep factors moderating clinical outcome. The wide array of secondary outcome measures, including emotion regulation, anxiety and depressive symptoms, quality of life and daytime functioning, allows us to compare results with currently running studies testing the effect of iCBT-I in other psychiatric disorders. Finally, due to the accessibility of the studied intervention, any positive findings of our RCT can be readily implemented in clinical practice.

The study also has some potential limitations. First, there is the risk of drop-out. The trial's thorough assessments may present a significant burden for patients with BPD (traits) that already suffer from severe emotional and behavioral symptoms and sleep problems. Especially patients in the control condition may be prone to drop out, as between T0 and T1, they are not actively engaged in a therapeutic process. We aim to reduce the risk of drop out by submitting patients in the control group to a somewhat active control condition by keeping a daily sleep diary. To prevent too much of a therapeutic effect, no feedback will be given, which in itself entails the risk of patients experiencing the sleep diary as too burdensome. Patients in the iCBT-I condition may experience sleep restriction as burdensome due to the possible temporary increase in emotional symptoms resulting from a lack of sleep. In both groups we aim to minimize drop-out by offering a central contact person to support them throughout the trial. Additionally, patients assigned to the control condition will be offered identical treatment of remaining sleep problems after T2. A second limitation is that a sleep diary-keeping waitlist control condition results in less attention from a professional compared to the iCBT-I condition (although our control condition is more active than a pure waitlist condition). This represents a non-specific, treatment-independent factor that may hinder interpretation of the findings. Finally, we base our power calculation on a meta-analysis of studies investigating the (added) effect of iCBT-I in patients with insomnia and other comorbid psychiatric disorders than BPD (e.g. MDD and PTSD) (Wu et al., 2015). Although BPD represents an archetypical emotion dysregulation disorder, which hypothetically would benefit profoundly from improving (the emotion regulatory function of) healthy sleep, the estimate of the effect size remains indirect, since no studies of iCBT-I in BPD exist.

To conclude, iCBT-I as add-on treatment for patients suffering from both BPD and sleep problems may be a promising way to alleviate BPD symptom severity directly and through improved internalization of subsequent standard BPD treatment.

## Abbreviations

BPD: Borderline personality disorder
BPDSI: Borderline Personality Disorder Severity Index
DERS-SF: Difficulties in Emotion Regulation Scale – Short Form
iCBT-I: Internet-based Cognitive Behavioral Therapy for Insomnia
ISI: Insomnia Severity Index
PD: Personality disorder
RMT-20: Rapid Measurement Toolkit-20
SCID-5-P: Structured Clinical Interview for DSM-5 Personality Disorders
STEPPS: Systems Training for Emotional Predictability and Problem Solving

## Trial status

The trial is open for inclusion.

## Funding

Funding by the Netherlands Brain Foundation (grant number: DR-2019-00319).

## CRediT authorship contribution statement

HvM and AvS obtained funding. All authors contributed to the study design and coordination. SvT wrote the manuscript, TvdZ, EvS, AvS, and HvM provided critical revisions. All authors read and approved the manuscript.

## Declaration of competing interest

The authors declare the following financial interests/personal relationships which may be considered as potential competing interests: AvS developed the iCBT-I treatment, but has no commercial interest. SvT, TvdZ, EvS, and HvM have no conflicts of interest to declare.

## Appendix A Supplementary data

Supplementary material Image 1
