# Supplementary material for: Guided internet-based cognitive behavioral therapy for insomnia in patients with borderline personality disorder: Study protocol for a randomized controlled trial
Source: Internet Interv. 2022 Jul 21;29:100563. doi: 10.1016/j.invent.2022.100563 (PMC9310106; doi:10.1016/j.invent.2022.100563)
Supplement: Supplementary file 1 — Supplementary material [file mmc1.docx]

**Supplementary material**

This supplementary material consists of a more detailed description of the internet-based cognitive behavioral therapy for insomnia (iCBT-I) treatment and additional baseline and outcome measures.

1. **iCBT-I (i-Sleep)**
   1. **Psychoeducation and sleep hygiene**

The first lesson consists of psychoeducation on (disturbed) sleep, and sleep hygiene rules about sleep promoting and sleep disturbing factors (e.g., bed times, napping, physical exercise, relaxing activities, alcohol, nicotine, and caffeine intake, screen time, and environmental factors such as bedroom temperature).

- 1. **Stimulus control and sleep restriction**

The second lesson introduces sleep restriction therapy: restrict the time in bed to the average amount of sleep (based on the first 7 nights of sleep diary data), with a minimum of 5 hours. Time in bed is restricted if the sleep efficiency (i.e., time spent in bed divided by time slept) is below 80%. Restricted bedtimes can be extended with 15 minutes if sleep efficiency is above 85% for a week. Patients are advised not to deviate from their bedtimes on weekend-nights and to avoid daytime sleeping (or at least restrict to naps of less than 30 minutes and before 3pm). Patients are encouraged to continue until they are satisfied with the amount of sleep, and advised to restrict their sleep again if sleep efficiency returns to below 80%.

Stimulus control focuses on not going to bed before feeling sleepy, using the bedroom for sleep and sex only, and getting up at night if patients are awake for longer than 15 to 30 minutes, only to return when feeling sleepy again.

- 1. **Relaxation and minimizing worrying**

The third lesson includes exercises to reduce intrusive thoughts and worrying and increase relaxation: (1) a daily 15-minute-window to write down thoughts and worries, (2) trying to stay awake at night, (3) blocking thoughts, (4) audio recorded muscle relaxation exercises.

- 1. **Tackling dysfunctional cognitions about sleep**

The fourth lesson includes cognitive strategies to tackle dysfunctional cognitions about sleep. The most common dysfunctional misconceptions about insomnia and its consequences are discussed, and strategies to challenge and change these cognitions are introduced (e.g., reappraisal, reattribution, attention shifting, hypothesis testing).

- 1. **Summary and relapse prevention**

The fifth (and final) lesson includes a summary of the content of the four previous lessons and evaluates any changes in patient’s sleep. The lesson also guides patients in developing a plan for the short and longer term future.

1. **Baseline measures**
   1. **Sleep quality**

Sleep quality is measured with the Pittsburgh Sleep Quality Index (PSQI); a self-report questionnaire consisting of 19 items, which is internally consistent, stable across time, and valid [1].

- 1. **Chronotype**

Chronotype is measured with the Munich Chronotype Questionnaire (MCTQ), a 17-item self-report questionnaire assessing typical daily sleep schedule, separately for workdays and free days, and asks patients to rate their chronotype on a scale of 0 (extremely early) to 6 (extremely late) [2]. An additional measure of chronotype is the Reduced Morningness-Eveningness Questionnaire (rMEQ), a shortened version of the 19-item Morningness-Eveningness Questionnaire (MEQ) [3], which contains five items. Total scores range from 4-25 and indicate one of the following preference groups: definitely morning type (score 22-25), moderately morning type (score 18-21), neither type (score 12-17), moderately evening type (score 8-11), or definitely evening type (score 4-7). The rMEQ has good convergent validity and high internal consistency, and is stable across time [4].

- 1. **Sleep disorders other than insomnia**

A questionnaire assessing symptoms of sleep disorders other than insomnia is also used, to look into possible narcolepsy, circadian sleep rhythm disorder, parasomnia, sleep breathing disorder, and periodic limb movements disorder/restless leg syndrome [5].

1. **Additional outcome measures at T0, T1, and T2**
   1. **Quality of life**

Quality of life is assessed with two different measures. First, the EuroQol-5D-5L (EQ-5D-5L) consists of five dimensions to assess mobility, self-care, daily activities, physical pain/discomfort, and emotional mood [6]. The EQ-5D-5L is an instrument with good reliability and both discriminant and convergent validity in populations with personality disorders [7]. Second, the Mental Health Quality of Life – 7 Dimensional (MHQoL-7D) aims to assess quality of life in people with mental health problems specifically [8]. The questionnaire consists of seven dimensions, rated on a scale ranging from 0 (very dissatisfied) to 3 (very satisfied), assessing self-image, independence, mood, relationships, daily activities, physical health, and hope. The MHQoL-7D has high internal consistency and test-retest reliability, and construct and discriminative validity [8].

- 1. **Positive mental health**

Because the absence of mental health complaints is not the same as the presence of positive mental health, we use the Positive Mental Health scale (PMH) to measure positive mental health aspects [9]. The PMH consists of nine items rated on a Likert scale ranging from 1 (not true) to 4 (true) with higher scores indicative of more positive mental health. The PMH is proven to have high internal consistency, good retest-reliability, good convergent and discriminant validity, and to be sensitive to therapeutic change [9].

- 1. **Life satisfaction**

Life satisfaction will be measured with Cantril’s ladder of life, which is a 2-item scale on which respondents rate their life on a ladder ranging from 0 (the worst possible life for you) to 10 (the best possible life for you). They do this for the present moment and for the expected situation in 5 years [10]. Cantril’s ladder has been shown to have adequate reliability and validity [11].

- 1. **Treatment evaluation**

Treatment evaluation questions are excerpts from the RE-AIM framework (i.e., Reach, Effectiveness, Adoption, Implementation and adherence, Maintenance of treatment effects) [12]. At T1, patients are asked to rate 1) their satisfaction with i-Sleep, 2) their satisfaction with the online guidance of i-Sleep, and 3) how helpful the different components of i-Sleep have been for improving their sleep. There is also room for patients to give suggestions on how to improve i-Sleep and/or its guidance. At T2 patients will be asked 1) to what extent they carried out the different components of i-Sleep after completion of the treatment, and 2) whether they initiated other activities to improve their sleep after the completion of i-Sleep. In addition, we keep track of the adherence to i-Sleep through the amount of completed sessions. Adherence to i-Sleep can be considered complete (4 or 5 sessions completed), partly complete (2 or 3 sessions completed), or not complete (less than 2 sessions completed).

**References**

[1] Buysse DJ, Reynolds CF, Monk TH, Berman SR, Kupfer DJ. The Pittsburgh sleep quality index: A new instrument for psychiatric practice and research. Psychiatry Res 1998;28:193–213. https://doi.org/10.1016/0165-1781(89)90047-4.

[2] Roenneberg T, Wirz-Justice A, Merrow M. Life between clocks: Daily temporal patterns of human chronotypes. J Biol Rhythms 2003;18:80–90. https://doi.org/10.1177/0748730402239679.

[3] Horne JA, Ostberg O. A self-assessment questionnaire to determine morningness-eveningness in human circadian rhythms. Int J Chronobiol 1976;4:97–110.

[4] Adan A, Almirall H. Horne & Östberg morningness-eveningness questionnaire: A reduced scale. Pers Individ Dif 1991;12:241–53. https://doi.org/10.1016/0191-8869(91)90110-W.

[5] Hombali A, Seow E, Yuan Q, Chang SHS, Satghare P, Kumar S, et al. Prevalence and correlates of sleep disorder symptoms in psychiatric disorders. Psychiatry Res 2019;279:116–22. https://doi.org/10.1016/j.psychres.2018.07.009.

[6] Herdman M, Gudex C, Lloyd A, Janssen M, Kind P, Parkin D, et al. Development and preliminary testing of the new five-level version of EQ-5D (EQ-5D-5L). Qual Life Res 2011;20:1727–36. https://doi.org/10.1007/s11136-011-9903-x.

[7] Janssen MF, Pickard AS, Golicki D, Gudex C, Niewada M, Scalone L, et al. Measurement properties of the EQ-5D-5L compared to the EQ-5D-3L across eight patient groups: A multi-country study. Qual Life Res 2013;22:1717–27. https://doi.org/10.1007/s11136-012-0322-4.

[8] van Krugten FCW, van Busschbach JJ V, Versteegh MM, Hakkaart-Van Roijen L, Brouwer WBF. The Mental Health Quality of Life Questionnaire (MHQoL): Development and first psychometric evaluation of a new measure to assess quality of life in people with mental health problems. Qual Life Res 2021. https://doi.org/10.1007/s11136-021-02935-w.

[9] Lukat J, Margraf J, Lutz R, Der Veld WM, Becker ES. Psychometric properties of the positive mental health scale (PMH-scale). BMC Psychol 2016;4:1–14. https://doi.org/10.1186/s40359-016-0111-x.

[10] Kilpatrick FP, Cantril H. Self-anchoring scaling: A measure of individuals’ unique reality worlds. J Individ Psychol 1960;16:158–73.

[11] McIntosh CN. Report on the construct validity of the temporal satisfaction with life scale. Soc Indic Res 2001;54:37–56. https://doi.org/10.1023/A:1007264829700.

[12] Glasgow RE, Vogt TM, Boles SM. Evaluating the public health impact of health promotion interventions: The RE-AIM framework 1999;89:1322–7. https://doi.org/10.2105/AJPH.89.9.1322.
